# Analysis of Occlusion Effects for Map-Based Self-Localization in Urban Areas

**DOI:** 10.3390/s21155196

**Published:** 2021-07-31

**Authors:** Yuki Endo, Ehsan Javanmardi, Shunsuke Kamijo

**Affiliations:** 1 Department of Information & Communication Engineering, Graduate School of Information Science and Technology, The University of Tokyo, Tokyo 153-8505, Japan; endou@kmj.iis.u-tokyo.ac.jp; 2 The Institute of Industrial Science (IIS), The University of Tokyo, Tokyo 153-8505, Japan

**Keywords:** autonomous driving, self-localization, LiDAR, point cloud map, urban, occlusion

## Abstract

A high-definition (HD) map provides structural information for map-based self-localization, enabling stable estimation in real environments. In urban areas, there are many obstacles, such as buses, that occlude sensor observations, resulting in self-localization errors. However, most of the existing HD map-based self-localization evaluations do not consider sudden significant errors due to obstacles. Instead, they evaluate this in terms of average error over estimated trajectories in an environment with few occlusions. This study evaluated the effects of self-localization estimation on occlusion with synthetically generated obstacles in a real environment. Various patterns of synthetic occlusion enabled the analyses of the effects of self-localization error from various angles. Our experiments showed various characteristics that locations susceptible to obstacles have. For example, we found that occlusion in intersections tends to increase self-localization errors. In addition, we analyzed the geometrical structures of a surrounding environment in high-level error cases and low-level error cases with occlusions. As a result, we suggested the concept that the real environment should have to achieve robust self-localization under occlusion conditions.

## 1. Introduction

Self-localization is a crucial technology to realize various tasks in autonomous driving, and research has been conducted to achieve stable operations in various environments. One way of self-localization is simultaneous localization and mapping (SLAM) [1,2], which estimates the 3D structure and trajectory sequentially based on sensor observations, such as laser imaging detection and ranging (LiDAR) scans [3]. Since SLAM estimates a state based on a previous estimation, it suffers from cumulative errors [2]. Loop closure, which optimizes the trajectory using loop information, can overcome the cumulative error problem [4,5] in specific cases, but it does not work well when there are few loops in the environment or when the distance to one loop is too long.

Alternatively, a method utilizing a pre-built high-definition (HD) map solves this problem. HD maps provide highly accurate information about the structure of the environment for self-localization, facilitating self-localization. Registration is one such method [6,7,8,9], which estimates a geometric transformation to match a sensor observation to a prebuilt map (e.g., a LiDAR scan to a point cloud map). HD map-based self-localization methods are known to be more accurate than SLAM methods because the estimated trajectory is ensured to be consistent by the consistency of the map.

One source of error in map-based self-localization is the ambiguity of the map structure to determine the self-localization results. Javanmardi et al. proposed factors that measure the map’s information content for self-localization [10,11]. The empirically defined factors enable us to estimate the self-localization error from a point cloud map before driving. For example, structures around an intersection are almost always roads and do not include enough distinctive structures for self-localization, resulting in ambiguous self-localization results.

**Figure 1:**
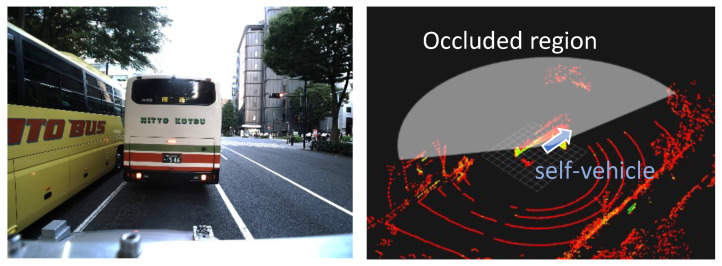
An example of occlusion in Shinjuku, Tokyo, Japan. (**Left**) The image taken from the self-vehicle. (**Right**) An overview of the LiDAR scan. The white colored area of the right image indicates the occluded regions by human annotation.

Another source of error in map-based self-localization is a discrepancy between observations and the map due to obstacles, such as large vehicles, especially in urban environments. Figure 1 shows an example of an occlusion situation in an urban area, Shinjuku, Tokyo, Japan.

Half of the LiDAR scans are occluded in this situation, resulting in insufficient observation of environmental information, and this would cause significant self-localization error.

However, while most existing self-localization studies have evaluated their method as one trajectory of the result [1,8,12], significant temporary errors caused by occlusion have not been investigated much. Occlusions of distinctive structures reduce the original information content from the map, resulting in unstable self-localization, but the analysis based on the factors by Javanmardi et al. was carried out in a static environment.

Therefore, this paper investigated how occlusions affect self-localization errors for each location in a real environment, especially in an urban environment. Synthetically generated obstacles in the real environment enabled the self-localization evaluations in various occlusion patterns, such as high-level (higher than 80%) and middle-level (from 40% to 60%) occlusions. Furthermore, we carried out information content-based analysis to reveal why the self-localization accuracy varies in specific occlusion cases and locations. In conclusion, we discuss what kinds of surrounding structures are suitable for accurate self-localization estimation even with many obstacles regarding information content-based analysis.

This paper is organized into the following sections. First, Section 2 introduces related works to our research, such as self-localization methods and self-localization evaluations. Next, Section 3 explains how to carry out our experiments with synthetically generated obstacles. Then, Section 5 evaluates how much the error in self-localization is increased on average by occlusion of the LiDAR scan. In addition, case studies are presented for cases where the error was significant, especially for low occlusion, and for cases where the error did not increase much, for high occlusion. In the next section, the structure of the surrounding environment is compared based on some criteria when the self-localization error is relatively high and low.

## 2. Related Work

### 2.1. Map-Based Self-Localization

One of the map-based self-localization methods is the registration algorithm. The registration algorithm optimizes a sensor pose that best matches the LiDAR scan to the map. For example, the iterative closest point (ICP) finds the nearest point of the map for each observation point and then solves the least-squares fitting between the pair of points [6]. On the other hand, the normal distribution transformation (NDT) defines a normal distribution based on the point cloud in each cell that divides the space into certain intervals. Then, the NDT solves the likelihood maximization problem of the LiDAR scan to the defined normal distribution (ND) map [7,13,14]. The ND map achieves a smaller capacity than the point cloud map used in the ICP.

On the other hand, some methods tackle the problem of the accuracy degradation of self-localization caused by occlusion [15,16,17,18]. Observation models for dynamic objects were built by Akai et al., resulting in robust self-localization even in dynamic environments. However, their experiments were not conducted in dense urban areas, so they did not consider large occlusions such as buses and trucks. Furthermore, the results were evaluated using average errors, ignoring suddenly occurring errors due to such occlusions.

Our study evaluated the effect of various patterns of occlusion on the self-localization error against obstacles. Our pseudo-occlusion in the real environment can generate more than 80% occlusion, which is rare in real space. We investigated the average error over the entire path and the cause of errors by location and occlusion.

### 2.2. Self-Localization Evaluation

The result that map-based self-location estimation leads to various uncertainties for locations was shown by Akai et al. in a rural environment [19]. To explain such uncertainties by a map structure, Javanmardi et al. listed factors for a map that express the information content that would determine the self-localization error [10,11]. In their analysis, the weighted sum of these factors from the map allowed estimating the self-localization error to some extent before driving. For example, the *feature sufficiency* measures the number of features in the map: lines, planes, and other scattered objects (called 1D, 2D, and 3D) [20]. As their result, 1D and 3D features positively affected self-localization, but the self-localization accuracy was degraded when many 2D features were included in the surroundings. On the other hand, the *feature layout* measures the extent of surrounding objects. If surrounding objects are clustered in one direction from the self-vehicle viewpoint, the self-localization estimation will be uncertain about the direction toward the cluster. This feature layout formulation is similar to the geometrical dilution of precision (GDOP) [21], which measures the spread of satellites in the global navigation satellite system (GNSS). However, they treated a map as static, but occlusion should reduce the positive effects of factors and decrease the self-localization accuracy in practice, especially in dense urban environments.

In contrast, this study analyzed such factors and self-localization error in dynamic environments. Many results using various occlusion patterns enabled us to understand the cause of self-localization errors due to specific occlusion patterns and find suitable environment structures for self-localization even in urban environments.

## 3. Synthetic Occlusion-Based Self-Localization Evaluation

Many buses and trucks cause large occlusions of LiDAR scans, resulting in drastic degradation of self-localization accuracy in urban environments, even in a location suitable for map-based self-localization. It is necessary to consider the static map structure and the effect of the degree from obstacles simultaneously to evaluate the self-localization accuracy in real environments correctly. We utilized synthetically generated obstacles in a real environment for the evaluation. Various patterns of generated occlusion enabled us to analyze self-localization from several angles.

### 3.1. Synthetic Obstacle Generation

In the evaluation, the obstacles were generated randomly in real environments. Because an obstacle should be placed on a lane, we annotated lane regions beforehand. Then, an obstacle was generated on a lane with some random vibration positions and orientations.

**Figure 2:**
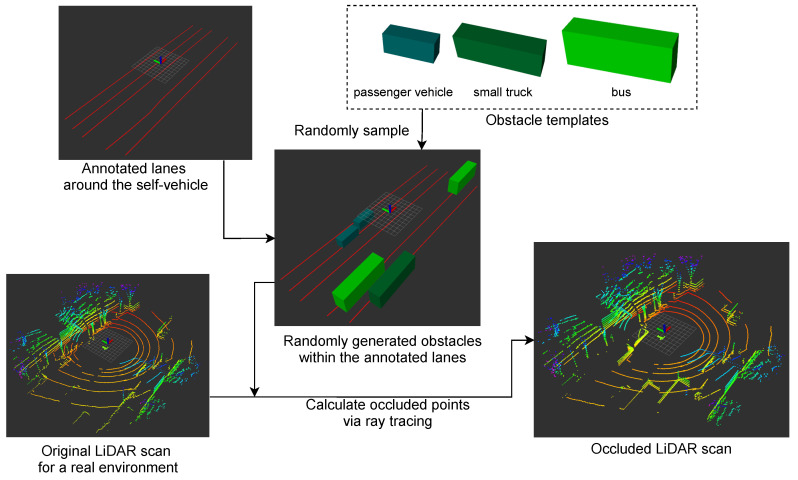
Process of occlusion generation. Note that the generation process was carried out on a real environment to simulate actual occlusion situations.

Figure 2 shows the flow of the obstacle generation process. First, one obstacle was randomly selected from the obstacle templates, including a passenger vehicle, a small truck, and a bus. Second, an obstacle position was sampled in the extracted lane region within a specific range from the self-vehicle. The line direction at the sampled position was regarded as the obstacle direction. Third, the position and the direction were each slightly varied by a random variable sampled from a uniform distribution. Then, the above generation process for a single obstacle was repeated as many times as the number of obstacles we wanted to generate. Finally, the occluded LiDAR scan caused by the generated obstacles was calculated via ray tracing. Since the generation process was random, self-localization using the occluded LiDAR scan was repeated several times in our experiments.

### 3.2. Evaluation with Multiple Initial Guesses

The self-localization accuracy highly depends on the initial guess at each time step since the optimization of registration algorithms is based on iterative parameter updates.

**Figure 3:**
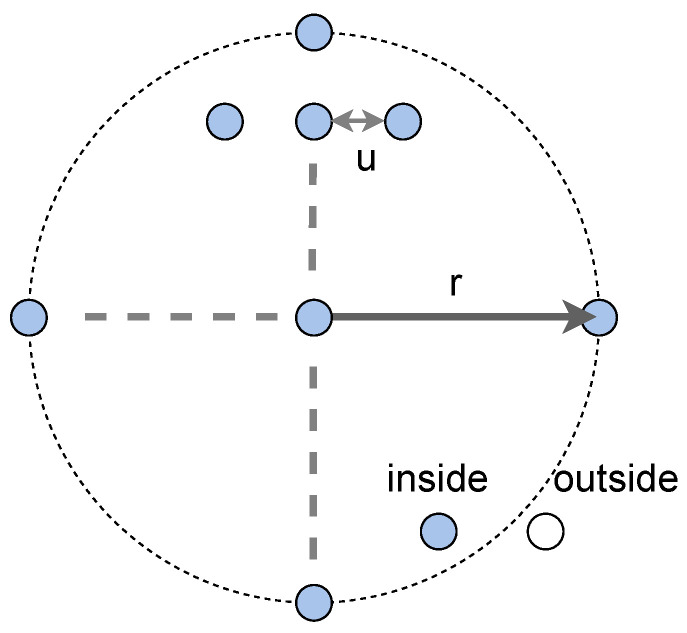
The initial guesses of the evaluation for each position. The blue colored circles in the range *r* (m) were used for the evaluation.

The evaluation using multiple initial guesses was carried out in our experiments to compare self-localization accuracy independently to initial guess accuracy. The self-localization results were estimated from predefined multiple initial guesses. The results were then summarized as a fair evaluation criterion for the initial guess uncertainty. The initial guesses for a certain time step were placed around the ground truth position. The placement of the initial guesses for a certain time is shown in Figure 3, where *r* is the radius of the initial guess range and *u* is the interval.

### 3.3. Evaluation Metric

As one of the metrics for our evaluation, an average pose error $e_{t}$ was used for a time step *t*. The definition of $e_{t}$ is as the following equation:

(1) $$e_{t}:=\frac{1}{\left|I\right|}\sum_{i\in I}{∥p_{t}^{\left(i\right)}-{\hat{p}}_{t}∥}_{2}$$

where |I| is the number of the initial guesses I, $p_{t}^{\left(i\right)}$ is the self-localization result from the initial guess *i*, and ${\hat{p}}_{t}$ is the ground truth pose at time *t*. Furthermore, ${∥\cdot ∥}_{2}$ calculates the $L^{2}$ norm. Typically, the average pose error increase $\Delta e_{t}$ is used to measure the increase in self-localization error from the original result to the result with synthetic occlusion as follows:

(2) $$\Delta e_{t}={\tilde{e}}_{t}-e_{t}$$

where ${\tilde{e}}_{t}$ is the average pose error increase with synthetic occlusion.

As an evaluation of the entire trajectory, we used the average of the $\Delta e_{t}$ over the time set T, that is $\Delta e=\sum_{t\in T}\Delta e_{t}$. Furthermore, the convergence ratio $\mu_{d}$ was used as a metric for the experiments. The convergence ratio $\mu_{d}$ represents how many estimations from the multiple initial guesses converged within a specific range to the ground truth positions. It is defined as the following equation:

(3) $$\mu_{d}:=\frac{1}{\left|T\right|}\sum_{t\in T}\frac{1}{\left|I\right|}\sum_{i\in I}𝟙\{∥p_{t}^{\left(i\right)}-{\hat{p}}_{t}{∥}_{2}\leq d\}$$

where $p_{t}^{\left(i\right)}$ and ${\hat{p}}_{t}$ are represented in the map coordinate system in terms of the X, Y, and Z coordinates, the roll angle, yaw angle, and pitch angle (the unit of the coordinates is meters, and the angle is in radians).

In all experiments, we performed self-localization with ten different patterns of occluded LiDAR scans for a certain number of obstacles for each time step. The number of occlusions was then changed to 9 patterns (5, 7, 9, 11, 13, 15, 17, 19, 21, 23, and 25), and the process was repeated. Namely, the self-localization was run 10×9×|I|×|T| times for a path.

## 4. Experimental Settings

This section describes the preliminary settings for evaluating the occlusion effects on self-localization, such as the experimental area, experimental vehicle, point cloud map generation, and definitions.

### 4.1. Experimental Area

**Figure 4:**
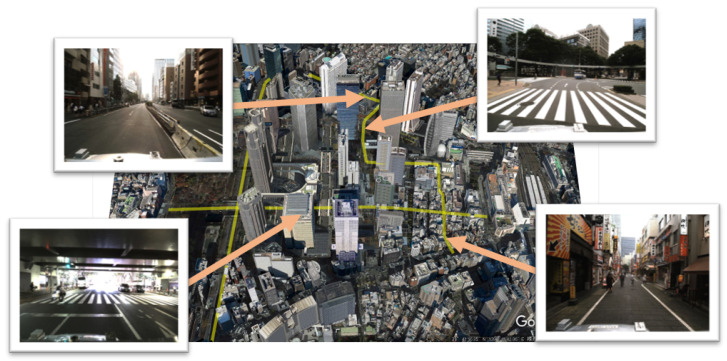
The appearance of the environment. The aerial photograph and images taken from the vehicle are shown for each notable location. They include trees, skyscrapers, and various road facilities.

All the experiments were conducted in Shinjuku, Tokyo, Japan, one of the most crowded urban environments. The experiments’ paths were more than 7.0 km. Figure 4 shows the appearance of our experimental paths.

The environment around the paths included trees, skyscrapers, and various road facilities. Furthermore, the environment had narrow roads and wide roads. GNSS-based self-localization is challenging in such urban environments because skyscrapers block signals from satellites and have multipath effects [22,23]. On the other hand, map-based self-localization methods can utilize the distributed geometrical structures of buildings and achieve high performance in some areas. However, there are many places where self-localization is difficult depending on the arrangement of the structures [11].

### 4.2. Experimental Vehicle

**Figure 5:**
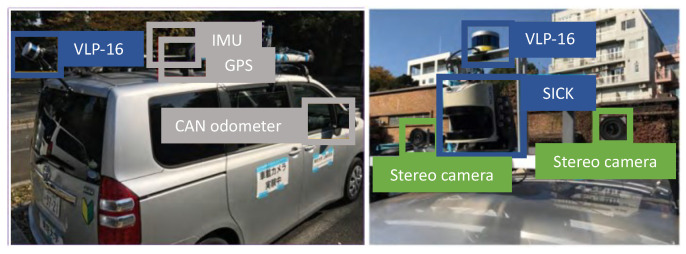
Experimental vehicle. Two sixteen-layer laser scanners (VLP-16) and one single-layer laser scanner were mounted for the point cloud map generation. One of the VLP-16s was used for self-localization in our experiments. Furthermore, the single-layer scanner, LMS511, was used to collect the reflection intensities of the LiDAR scans.

Figure 5 shows our experimental vehicle used for point cloud map generation and self-localization.

Two sixteen-layer laser scanners (Velodyne LiDAR VLP-16) and one single-layer laser scanner (SICK LMS511) were utilized to generate a point cloud map. For the self-localization process with a generated point cloud map, only one horizontally mounted VLP-16 was used. In the generation process of a point cloud map, the horizontally mounted VLP-16 scanner was used for self-localization, and the one mounted at an angle of 75 degrees was used to generate a point cloud. Besides these scanners, the LMS511, a single-layer scanner, was used to collect the reflection intensities of the LiDAR scans to perform a calibration for the generated map [24]. The calibration method was based on matching aerial imagery with road marking information obtained from the point cloud map’s reflection intensity and made the point cloud map globally consistent.

### 4.3. Ground Truth Definition

Many studies related to self-localization face the problem of how we should define the ground truth. Standard datasets for self-localization define the ground truth by using expensive differential GPS (DGPS) and real-time kinematic (RTK) GPS with modern SLAM methods [12,25]. However, achieving accurate positioning with these devices is challenging in dense urban environments such as Shinjuku, Tokyo, Japan, because of the skyscrapers.

A grid search-based optimization for point cloud registration was carried out to define the ground truth reasonably. The global optimum of registration should be rather accurate on a global coordinate system since the point cloud maps used in our experiments were calibrated with aerial imagery. First, a rough trajectory was estimated using the NDT, a registration algorithm, with a grid size of 1.0 m from a pre-defined initial guess at the start time. Then, for each pose of the estimated trajectory, the registration score was calculated every 0.05 m within 0.5 m in the x and y directions from the pose, and the pose with the highest score (the global optimum) was defined as the ground truth pose.

Since our point cloud maps were guaranteed to be globally consistent by the map calibration process using aerial imagery, as described in the previous section [24], the ground truth poses were also guaranteed to be highly accurate.

### 4.4. Self-Localization Method

As the target self-localization method of our experiments, the normal distribution transformation (NDT) was adopted. NDT optimizes a transformation between a LiDAR scan and a map to maximize a score function that measures how well the scan fits the map. Let us give an overview of the NDT process.

First, as a preparation process before self-localization, a normal distribution (ND) map, a set of normal distributions, was defined from the point cloud map. The cubic cells that divided the 3D space at regular intervals *d* were determined and defined the set of normal distributions by the points contained in each cell. Second, as an estimation for each time, a transformation between the LiDAR scan and the ND map was optimized to solve a likelihood maximization problem. The problem was defined by using a score function s:SE(3)→R as the following equation.

(4) $$\underset{T_{lm}}{arg max}s\left(T_{lm}\right)$$

(5) $$s\left(T_{lm}\right)=\sum_{i=1}^{N}p\left(T\left(x_{i},T_{lm}\right)\right)$$

where $T:\left(R^{3},SE\left(3\right)\right)\to R^{3}$ is a transformation function to transform a scan point $x_{i}$ to a map coordinate system using a transformation matrix $T_{lm}\in SE\left(3\right)$. The function *p* is a function to measure likelihood for a point $x_{i}$ as the following equation:

(6) $$p\left({\tilde{x}}_{i}\right)=\exp\left(-\frac{{\left({\tilde{x}}_{i}-\mu_{i}\right)}^{T}\sum_{i}^{-1}\left({\tilde{x}}_{i}-\mu_{i}\right)}{2}\right)$$

where $\mu_{i}$ and $\sum_{i}$ are the parameters of a normal distribution defined from the map on which a transformed scan point ${\tilde{x}}_{i}$ lies.

The regular interval d=2.00 m was adopted for all the experiments.

### 4.5. Obstacle Definition

In our experiments, three types of vehicles acted as obstacles, as shown in Table 1. The size of these vehicles was simulated to represent common vehicles in Japan.

The bus was the largest vehicle in the obstacle candidates and could occlude half of LiDAR scan points. An example of occlusion by a bus is shown in Figure 1. Such occlusions often appear in real urban environments. Besides buses, passenger vehicles, the smallest obstacle on the list, appear many times in practice. Small occlusions by passenger vehicles sometimes cause significant self-localization errors because such obstacles occlude curbs, which can be a valuable feature for latitudinal positioning.

## 5. Self-Localization Evaluation Results with Occlusions

### 5.1. Determination of the Error Ranges of an Initial Guess Estimation

Initial guesses for map-based self-localization are usually estimated by combining estimation results from the inertial navigation system (INS) and the Global Navigation Satellite System (GNSS). To determine the *r* described in Section 3.2, we should consider two situations of initial guess estimation. First, when the uncertainty of the posterior distribution of the filtering process has become small, such as the vehicle has been driven for a certain amount of time, the estimated initial guess has a relatively high accuracy. On the other hand, just after the start of driving or just after it has been lost, a prior distribution in the filtering process is not well estimated and makes the error of initial guess estimation more significant. This paper focuses on the former case as an initial guess estimation.

Then, we determined the *r* when the initial guess estimation was relatively stable. The work of Gu et al. evaluated the accuracy of position estimation for a device that combined the INS and GNSS processing and their proposed positioning that took multipath into account using a 3D model of a building in Hitotsubashi, Tokyo, Japan [23]. According to the experimental results in the paper, the positioning error was 1.78 m on average for the device that combined INS and GNSS and 1.42 m on average for their proposed method. From these results, we can conclude that the deviation of the initial guess was about 2.00 m when the filtering was stable, and we adopted r=2.00 m.

We also set the distance between adjacent initial guesses *u* (m) to be 0.2 m.

### 5.2. Average Effects of Occlusion

**Figure 6:**
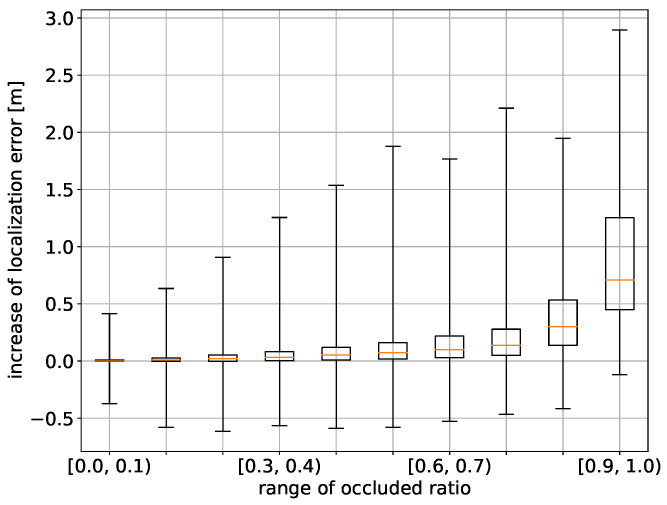
Effect of occlusion on self-localization error. The errors on 10 occlusion levels are shown. Each vertical line for an occlusion level shows the error range, and the box shows the interquartile range. Each orange line shows the median of the error.

First, let us look at the average effects of occlusions on self-localization error. Figure 6 shows the increases of self-localization error relative to the original error with different ranges of the occlusion ratio. The initial guess range was set to 2.0 m.

The average increase in the self-localization error rose with the increase of the occlusion ratio. Remarkably, there were cases where the self-localization error increased more than 1.5 m with an occlusion ratio of around 50%. Besides such cases, there were cases where the self-localization error did not increase much even at a high occlusion ratio of more than 80%. In the next section, we will see such cases individually and discuss the causes. On the other hand, some self-localization errors decreased with occlusion. This can be interpreted as occluding inferior features in an environment improved the self-localization accuracy.

**Figure 7:**
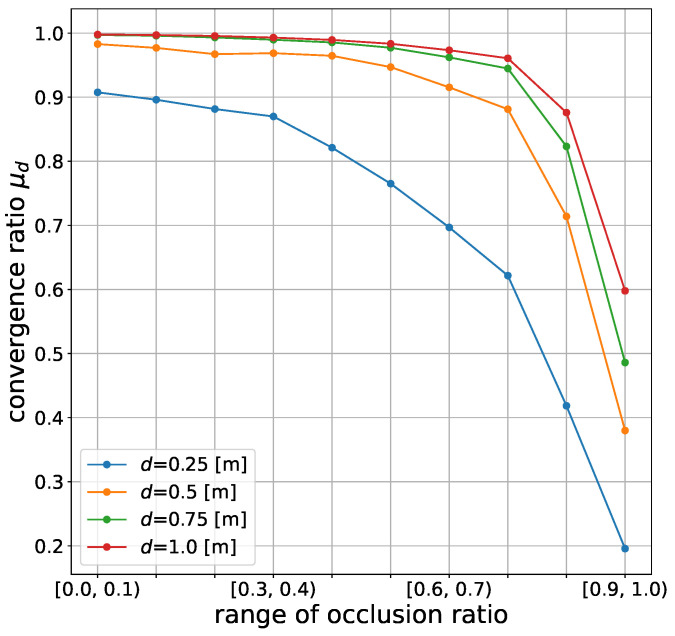
Effect of occlusion on the convergence ratio of the self-localization results at different convergence thresholds *d*. The convergence ratios on 10 occlusion levels are shown.

Figure 7 shows the convergence ratios of the self-localization results for different distances d=0.25,0.5,0.75,1.0.

The convergence ratio decreased with the increase of the occlusion ratios. The convergence ratio decreased significantly when the occlusion ratio exceeded 0.8. This result implies that self-localization would fail most of the time in highly occluded situations.

### 5.3. Case Study for Different Occlusion Cases

The result in Figure 6 shows that large variances in self-localization error increase with high occlusion ratios. This indicates that the self-localization error varies significantly depending on the occluded situations. Therefore, by the case studies, we evaluated what kind of occlusion caused large fluctuations in the self-localization estimation. Significantly, the scenario of high error under about half occlusion and the scenario of low error under high occlusion were considered.

#### 5.3.1. Scenario of High Error under Half Occlusion

**Figure 8:**
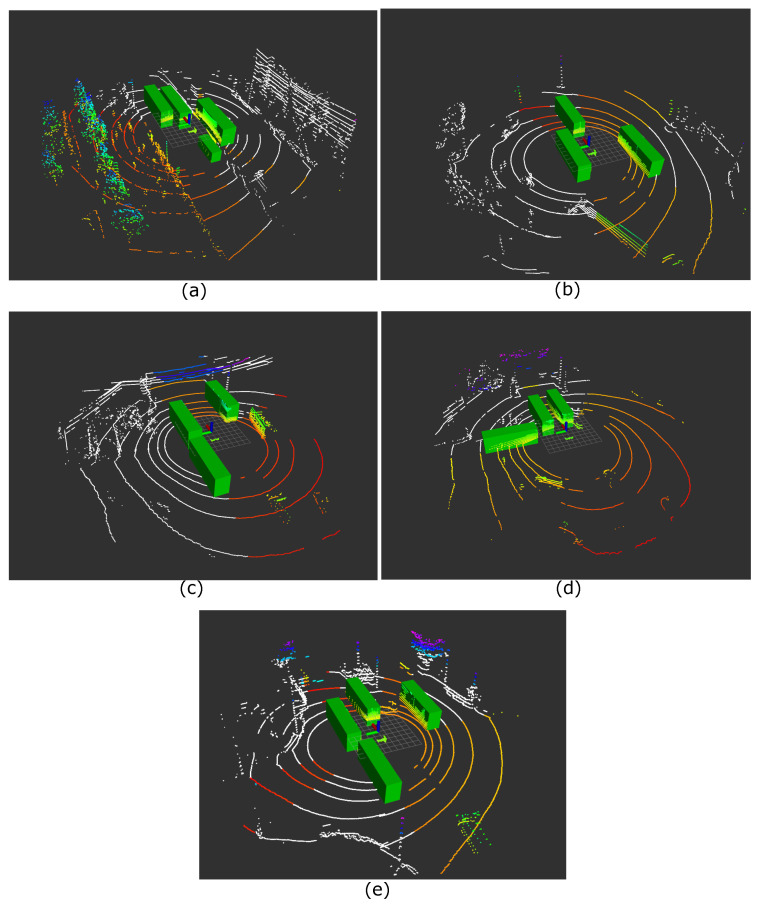
High self-localization error cases (**a**–**e**) with middle-level occlusion. The white colored points indicate invisible scan points due to obstacles. The color of the visible scan point changes from red to blue as its height increases.

We evaluated five error increase situations with a 0.4 to 0.6 occlusion ratio to see cases where the self-localization error varied greatly in half occlusion situations, which often occur in real environments. Figure 8 shows the appearance of the situations, and Table 2 shows the results of the self-localization estimation.

When the self-localization error became huge, even in middle-level occlusion, structures in the places seemed to be concentrated in specific directions. Huge errors occurred when such directions were occluded by large vehicles and there were enough features for successful self-localization in the observed regions. Notably, Situations (b)–(e) occurred at intersections. Intersections often have few structures and are considered to be vulnerable to occlusion because structures can be easily occluded, as was the situation.

**Figure 9:**
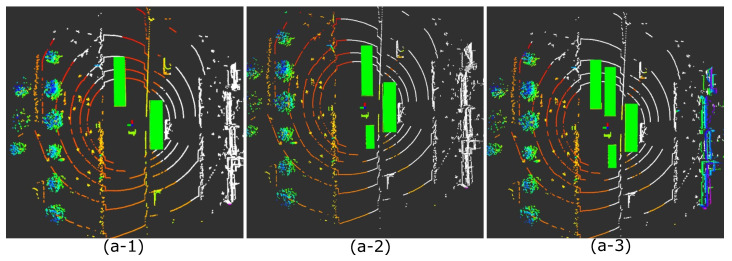
Occlusions from the bird’s-eye view when specific areas were made observable in the case of Figure 8a. The white-colored points indicate invisible scan points due to obstacles. The visible scan point changes from red to blue as its height increases. (**a-1**) A curb on the right side is visible,. (**a-2**) A pole and a curb in front on the left side are visible. (**a-3**) The walls of the buildings on the right side are visible.

Furthermore, the cause of the decrease in self-localization accuracy was analyzed for each occluded region in Figure 8a. Primarily, we divided the occluded scan points into three parts: the walls of buildings on the right, a pole and a curb in front on the left, and a curb on the right. Figure 9 shows how the scan was occluded from a bird’s-eye view when only one of the three target regions became observable. Table 3 shows the variation of the self-localization error in each case.

Some error reduction was verified when either the pole or the curb became observable, but a significant improvement was realized when the building wall became observable. This indicates that the building wall could be a vital feature for accurate self-localization in this case, but it was easily occluded even by a single bus, as Figure 8a.

#### 5.3.2. Scenario of Low Error under High Occlusion

**Figure 10:**
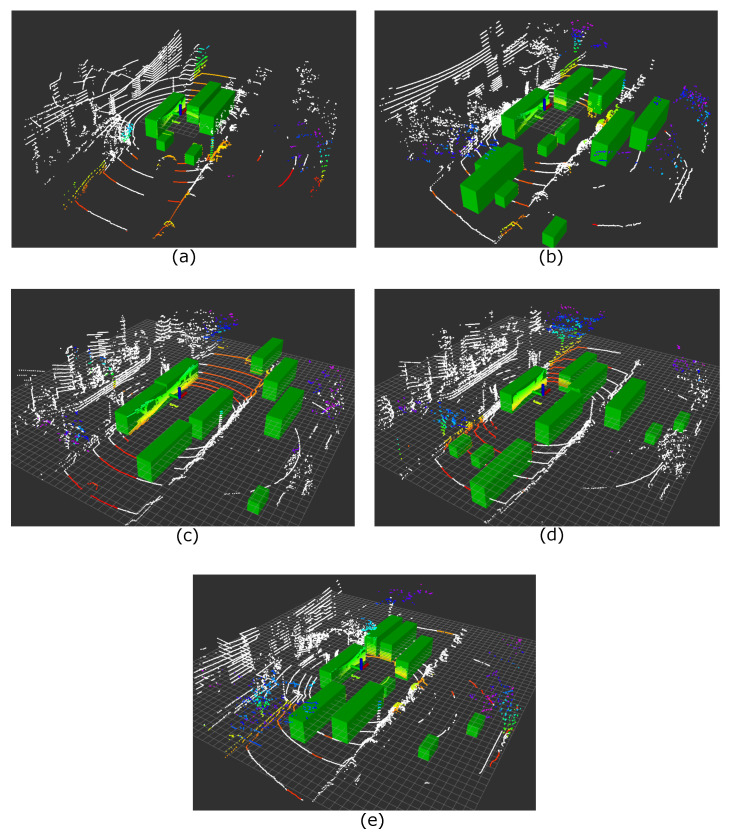
Low self-localization error cases (**a**–**e**) in high-level occlusion, above 80% occlusion. The white colored points indicate invisible scan points due to obstacles. The color of the visible scan point changes from red to blue as its height increases.

Next, we investigated another pattern of situations where the self-localization accuracy was almost the same as the original one, even with about 80% occluded scan points. As the previous scenario showed, five self-localization error increases were found. Figure 10 shows the different occlusions at the same location, and Table 4 shows the variation of its error. In all the situations, the error increased only from 0.063 m to 0.187, m even though 80.1% to 84.7% of the scan was occluded.

One possible reason is that the scans contained pole-like objects. It was considered that observing large structures such as walls was challenging. On the other hand, it was easy to observe small-sized structures such as pole-like objects or curbs in high-occlusion ratio situations. In addition, such small-sized structures have the capability to improve the self-localization accuracy, as shown by Javanmardi et al. [11]. On the other hand, walls can be occluded easily, and plane objects do not have such a capability for accurate self-localization. However, it is not clear that pole-like objects can help self-localization in highly occluded situations. This problem is discussed in the next section using the criteria of the latent map capability.

## 6. Information Content Analysis of Error Sensitivity

LiDAR-based self-localization accuracy highly depends on the map structure. For example, the self-localization accuracy may be worse when flat walls surround the self-vehicle. In contrast with walls, distinctive objects, such as poles and building edges, help self-localization accuracy. However, if distinctive objects are concentrated in one place, self-localization uncertainty remains in one direction. Furthermore, these objects can be occluded easily in such arrangements.

Javanmardi et al. proposed criteria to model information content that would determine self-localization errors from a point cloud map structure [10,11]. First, let us review the definition of the criteria, such as feature sufficiency and feature layout. Then, we will see the effect of the self-localization accuracy in dynamic environments with the criteria. Unlike the static analysis of self-localization error, proposed in [10,11], the effects of occlusion were analyzed based on the information content of a map.

### 6.1. Feature Sufficiency Criterion

Distinctive objects such as pole-like objects, building walls, and trees in the environment are known to be valuable features to solve the registration problem of matching a LiDAR scan to a point cloud map. The feature sufficiency criterion measures how many features are included in a map for three feature types. The three feature types are called 1D, 2D, and 3D features. The 1D, 2D, and 3D features represent a pole-like object, a planar object, and a spatially spread object, respectively. The original idea of the geometrical classification for a point cloud map was proposed by [20].

These features are defined for each cell in a fixed interval of space by the eigenvalues of the point cloud inside it. Note that such cells were already defined in the preparation phase of NDT. Let $\lambda_{1}\geq \lambda_{2}\geq \lambda_{3}\geq 0$ be the eigenvalues of a point cloud within a cell, and negative eigenvalues were assumed to be absolute values. Now, the squared eigenvalues are defined as follows.

(7) $$\sigma_{i}=\sqrt{\lambda_{i}}\;\;i=1,2,3$$

Using these squared eigenvalues, the criteria to determine feature types are defined as follows.

(8) $$a_{1D}=\frac{\sigma_{1}-\sigma_{2}}{\sigma_{1}},\;a_{2D}=\frac{\sigma_{2}-\sigma_{3}}{\sigma_{1}},\;a_{3D}=\frac{\sigma_{3}}{\sigma_{1}}$$

**Figure 11:**
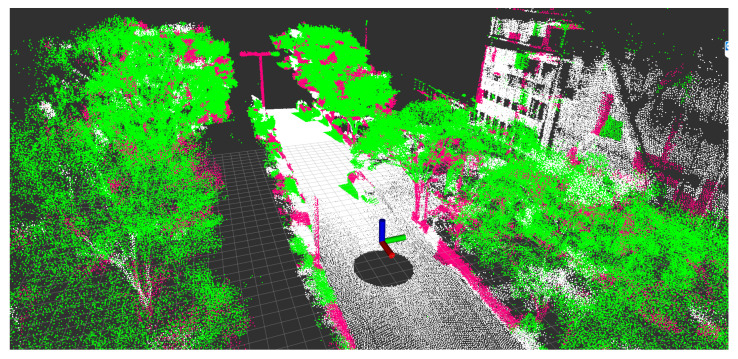
The appearance of 1D, 2D, and 3D features in an urban environment. Pink, white, and green represent 1D, 2D, and 3D features, respectively.

If $a_{1D}\geq a_{2D},a_{3D}$, the point set in a cell is defined as a 1D feature. In the same way, if $a_{2D}\geq a_{1D},a_{3D}$, the point set in a cell is defined as a 2D feature; otherwise, the point set in a cell is defined as a 3D feature. Figure 11 shows the appearance of 1D, 2D, and 3D features in an environment.

### 6.2. Feature Layout Criterion

Besides the feature sufficiency criterion, the feature layout criterion is vital as well. Let us look at an example of the effect of the feature layout on self-localization accuracy. If almost all features concentrate in one direction from the self-vehicle viewpoint, the self-localization result should have uncertainty towards that direction.

$F_{DOP}$ is a kind of the geometrical dilution of precision (GDOP) described in the GNSS. The PDOP is a value to measure how navigation satellites are spread. Similar to the idea, $F_{DOP}$ measures how the features are spread. Let $p_{i}=\left(x_{i},y_{i},z_{i}\right)$ be the center position of a cell *i*. $F_{DOP}$ is defined as follows:

(9) $$F_{DOP}=\frac{1}{\sqrt{\tilde{\sigma_{x}^{2}}+\tilde{\sigma_{y}^{2}}+\tilde{\sigma_{z}^{2}}}}$$

where $\tilde{\sigma_{x}^{2}}$, $\tilde{\sigma_{y}^{2}}$, and $\tilde{\sigma_{z}^{2}}$ are the variances of each coordinate. Higher $F_{DOP}$ indicates that the features is more distributed in the surrounding environment.

### 6.3. Factor-Based Evaluation of Self-Localization Error in Occlusion

This section analyzes what occlusions affect the self-localization error with the criteria described in the previous sections.

**Figure 12:**
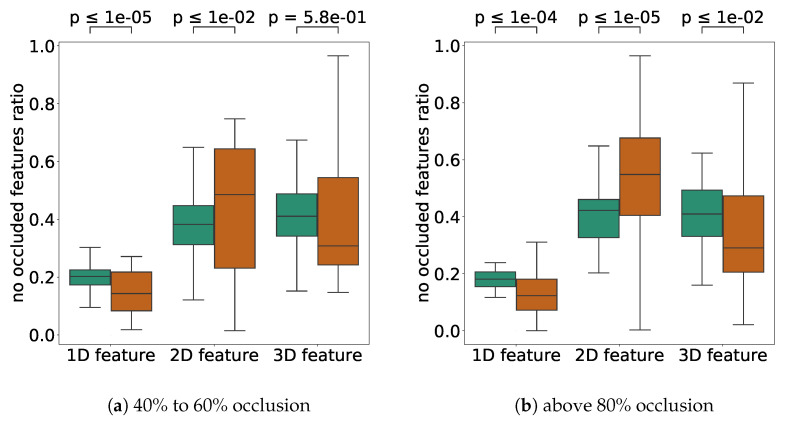
A comparison of the number of each nonoccluded feature for different self-localization error levels. A brown bar shows the result for a large error (≥1.0 m), and a green bar shows the result for a small error (0.1 m). (**a**) is the result for the scans with about 50% occlusion (40%≤ and <60%). (**b**) is the result for the scans with >80% occlusion. The *p*-values indicate the Welch T-test results for each pair of comparisons. The outlier values were removed in the figure.

First, we compared the ratio of features in the open area in high-error cases (more than 1.0 m) and low-error cases (less than 0.1 m) with about half of the occluded LiDAR scan points. The results are shown in Figure 12. The *p*-values for the application of Welch’s T-test are shown in the upper part of the figure to consider the difference in the number of samples. From the result Figure 12a, when the self-localization error was low, the proportion of 1D features was significantly high. Conversely, when the error was high, the percentage of 1D features was low, but the rate of 2D features was high. This result may reflect the fact that the error was higher near intersections such as Figure 8b–e in Figure 8, where only the road surface was visible because the features were occluded.

The same conclusions can be drawn for the 1D and 2D features for the high occlusion case. For this high occlusion ratio, the percentage of 3D features was significantly higher when the self-localization error was low. This result may mean that the presence of structures on the ground, such as curbs, as seen in Figure 10a,c,d,e, was essential for maintaining the self-localization accuracy at high occlusion ratios.

### 6.4. Places Where the Self-Localization Accuracy Is Affected by Occlusion

The previous section analyzed the variation in the self-localization error from surrounding unobstructed features. Depending on the original surrounding environment structures, some locations may be more susceptible to obstructions on average.

**Figure 13:**
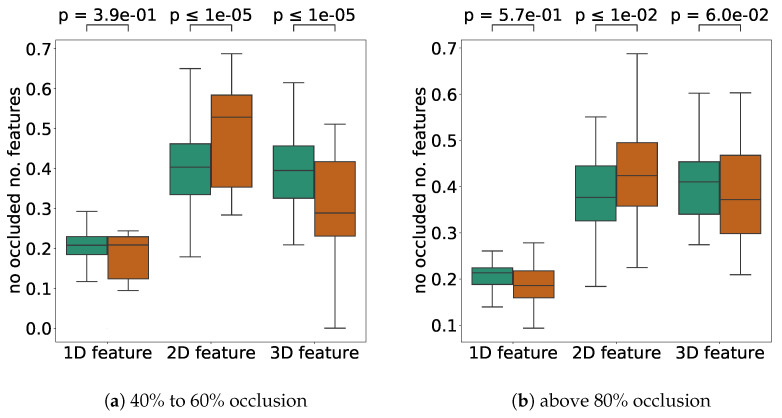
A comparison of the number of each feature for different self-localization error levels. A brown bar shows the result for a large error (≥1.0 m), and a green bar shows the result for a small error (0.1 m). (**a**) is the results for the scans with about 50% occlusion (40%≤ and <60%). (**b**) is the results for the scans with >80% occlusion. The *p*-values indicate Welch *T*-test results for each pair of comparisons. The outlier values were removed in the figure.

Figure 13 shows a comparison of the number of 1D, 2D, and 3D features on the map between high and low self-localization accuracy.

When about 50% of the LiDAR scan points were occluded (40%≤ and <60%), it can be seen that the self-localization error was higher with many 2D features. Since 2D features are planar and have less ability to determine the self-position in the direction parallel to the planar surface, the self-localization error tended to increase in places with many planar surfaces when the occlusion ratio was high. Cases of Figure 8b–e are examples of a location with a large number of 2D features and a small number of 1D and 3D features. In such a location, the error tended to increase due to occlusion. For the high occlusion cases, while the null hypothesis could not be significantly rejected for 1D and 3D, we can see that the error was affected by the occlusion when there were too many 2D features. On average, the 1D and 3D features should be sufficiently scattered in the environment to achieve robust self-localization against occlusion. In particular, in a real environment, most of the features are flat, such as the center of an intersection, and there are few spiky features in the surrounding area.

**Figure 14:**
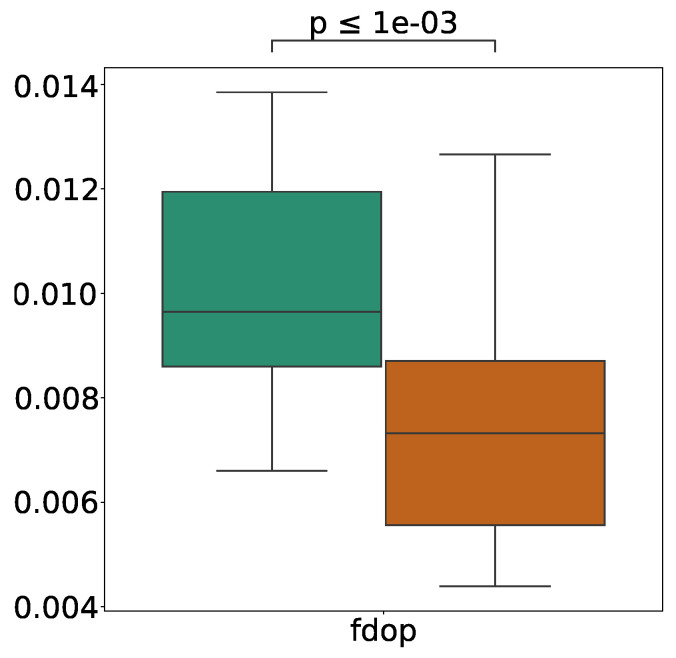
Comparison of $F_{DOP}$ for different error levels when the number of 2D features is less than the median and 80% of scan points are occluded. The outlier values were removed in the figure.

However, even if there are few 2D features, 1D and 3D features could be easily occluded if they were clustered in one place. Figure 14 shows the results of the $F_{DOP}$ comparison when the number of 2D features was below its median.

When the error was low, the value of $F_{DOP}$ was significantly high, i.e., the features were spread out. This result means that even if an environment has fewer 2D features (i.e., there are enough 1D and 3D features), it is not a suitable environment for self-localization if those features are concentrated at a single point. This result is consistent with the results for static environments [11]. In particular, in environments with low $F_{DOP}$, the probability of a single obstacle occluding the entire feature also increases, which may have caused such results.

In summary, an environment with few 2D features, i.e., planar objects, and a uniform distribution of 1D and 3D objects in the environment is considered to be an environment where stable self-positioning can be performed even under the influence of obstacles.

## 7. Conclusions

In this paper, the effects of obstacles on the map-based self-localization method were evaluated by using various patterns of synthetically generated occlusions. In particular, in urban areas, where many structures may help accurate self-localization without obstacles, the error increased by about 2.0 m with about 50% occlusion. On the other hand, there were cases where the accuracy of self-localization was not affected by the high-level occlusion ratio. In such cases, it was confirmed that small objects such as curbs and poles were observed through the occlusion. In addition, we experimentally showed that the self-localization accuracy tended to decrease in regions where there were many planar objects in the vicinity of the point cloud map, such as intersections, for both medium and high levels of occlusion. In addition, we showed that the self-localization accuracy tended to decrease when the objects in the surrounding environment were more spread out, even under the occlusion condition.

The conclusion is that objects that can be landmarks for self-localization are less likely to be observed under occlusion conditions in places where structures exist in a single location or intersections. In future urban development, it will be necessary to spread pole-shaped objects over the environment to be observed in such situations.

## Figures and Tables

**Table 1:** List of obstacles.

Type of Vehicle	Width (m)	Height (m)	Length (m)
passenger vehicle	1.8	1.5	3.0
small truck	1.7	2.0	4.7
bus	2.5	3.7	10.0

**Table 2:** Change in self-localization error with about 50% occlusion.

Case	Localization Error Increase (m)	Occlusion Ratio
(a)	1.981	0.530
(b)	2.236	0.598
(c)	1.507	0.565
(d)	1.230	0.400
(e)	0.812	0.507

**Table 3:** Change in self-localization error when a specific region is made observable.

Case	Pole	Curb	Wall	Localization Error Increase (m)	Occlusion Ratio
(a-1)	√			1.001	0.485
(a-2)		√		1.100	0.459
(a-3)			√	0.173	0.372

**Table 4:** Self-localization error increases with about an 80% occlusion ratio.

Case	Localization Error Increase (m)	Occlusion Ratio
(f)	0.063	0.825
(g)	0.187	0.841
(h)	0.112	0.803
(i)	0.144	0.847
(j)	0.083	0.801

## Data Availability

Not applicable.

## Abbreviations

The following abbreviations are used in this manuscript:

SLAM	Simultaneous localization and mapping
HD map	High-definition map
NDT	Normal distribution transformation
ICP	Iterative closest point
GNSS	Global Navigation Satellite System
GPS	Global Positioning System
INS	Inertial navigation system
LiDAR	Light detection and ranging
GDOP	Geometrical dilution of precision
FDOP	Feature dilution of precision
